# Effective engagement of a stakeholder advisory board in severe mental illness (SMI) research: A case study of a clinical trial to improve adherence among people with SMI and hypertension

**DOI:** 10.5430/ijh.v8n2p9

**Published:** 2022-07-01

**Authors:** Dafna Paltin, Jessica L. Montoya, Celeste Weise, Carla Conroy, Ethan E. Radatz, Kurt C. Strange, David J. Moore, Martha Sajatovic, Jennifer B. Levin

**Affiliations:** 1 SDSU/UC San Diego Joint Doctoral Program in Clinical Psychology, San Diego, California, USA.; 2 Department of Psychiatry, UC San Diego, San Diego, California, USA.; 3 Department of Psychiatry, Case Western Reserve University School of Medicine, Cleveland, Ohio, USA.; 4 Neurological and Behavioral Outcomes Center, University Hospitals of Cleveland Medical Center, Cleveland, Ohio, USA.; 5 Department of Psychiatry, University Hospitals of Cleveland Medical Center, Cleveland, Ohio, USA.; 6 Department of Family Medicine and Community Health, Case Western Reserve University School of Medicine, Cleveland, Ohio, USA.; 7 Department of Population and Quantitative Health Sciences, Case Western Reserve University School of Medicine, Cleveland, Ohio, USA

**Keywords:** Community Engagement, Bipolar Disorder, Medication Adherence, mHealth, Text Messaging, Implementation, RE-AIM framework, Blood Pressure

## Abstract

**Objective:**

Poor adherence to antihypertensive medication occurs in 50-80% of patients. An ongoing randomized controlled trial (RCT) is evaluating a personalized mobile-health intervention in poorly adherent hypertensive persons with bipolar disorder. To enhance efficacy, the ongoing trial elicited guidance from a Stakeholder Advisory Board (SAB) comprised of patients, family members, clinicians, and health system administrators. Our goal is to describe the formation, role, decision-making process, and key contributions of the SAB as a means of demonstrating meaningful community engagement in mental health research.

**Methods:**

Using models and measures from the field of implementation science, eleven SAB members convened across three meetings followed by quantitative surveys that assessed SAB member satisfaction and engagement during the meeting.

**Results:**

Significant suggestions from the SAB included 1) expanding inclusion/exclusion criteria, and 2) operationalizing remote implementation of the RCT. Primary study implementation challenges identified by the SAB were 1) participant difficulty engaging in the mHealth intervention, and 2) identification of procedures for monitoring participant adherence to the RCT protocol and contacting under-engaged participants. Quantitative surveys indicated that all SAB members believed that the objectives of the meetings were clear, perceived that they were able to participate in the discussions, and that they were heard.

**Conclusions:**

Increasing evidence demonstrates the feasibility of engaging with SABs in clinical research and that this process improves intervention design, increases participant engagement, reduces mental health-related stigma, and produces more effective implementation strategies. We encourage future investigators to use an implementation science framework in partnership with SABs to refine their proposed interventions and improve clinical outcomes

## 1. Introduction

The benefits to involving stakeholders in SMI research are increasingly being recognized. Stakeholders generally refer to people with a variety of lived experience and personal interest in a given patient population and is inclusive of patients, family members, community members, health services providers, and clinicians. Stakeholders provide unique insights and valuable perspectives that can be utilized to support research efforts. It is clear that involving stakeholders is important for quality and impactful research [1-5], however, there is limited information on how to engage stakeholders (e.g., formation of groups, roles, decision-making processes) or the specific value that stakeholders bring to research projects. The current study focuses on methods for effective engagement with a Stakeholder Advisory Board (SAB) and key contributions of stakeholders in research to improve community-engaged SMI research.

Current best practices for optimal stakeholder engagement are rooted in the foundational principles of respect, equity, trust and empowerment [4]. Best practices to support stakeholder engagement in research include: 1) research training and education for patients and researchers, 2) clarification of roles and expectations for research team and stakeholders, 3) compensation for SAB participation, 4) evaluation and reinforcement of engagement, 5) regular meetings with open communication, and 6) involving stakeholders early in the research process with regular acknowledgment of their contributions [4]. Many studies include stakeholders in their design [6-10] and some discuss stakeholder involvement without explicit detail about how and when stakeholders were engaged [1, 5, 11-13], but few include instructions or best practices for how to work with stakeholders, manage their contributions, or the shared decision-making process between stakeholders and research teams [2, 3]. There is a particular gap in the SMI literature regarding how to approach and implement stakeholder feedback across different stages of clinical trials.

Despite many potential benefits, there are significant challenges with stakeholder engagement in research. Identifying a representative sample and building trusting relationships is time consuming for both researchers and stakeholders alike. This justifies defining the purpose, roles and expectations of stakeholders as early in the process of study design as possible. Additionally, limited communication between scientific and clinical teams can place undue stress on stakeholders [2], so regularly scheduled meetings can be essential. Integrating input from stakeholders into an active study can also present unique challenges, especially when requiring IRB approval for any proposed changes, which can delay a study’s timeline. Specific to SMI research, there is an overall lack of consistency in frameworks or model methodologies for engaging with stakeholders, which makes results difficult to compare. Even less is known about the challenges of stakeholder engagement in studies addressing multi-morbidities, such as the population of interest (i.e., people with bipolar disorder type I or type II and hypertension) in our present RCT.

The present study is an ongoing randomized controlled trial (RCT) evaluating a personalized, mobile health, patient-centered medication adherence intervention in poorly adherent persons with bipolar disorder (type I or II) and hypertension. To ensure that the proposed adherence intervention meets the needs of patients, clinicians, and clinical operational workflows, the ongoing trial elicited iterative input from a stakeholder advisory board (SAB) comprised of patients, family members, clinical providers, and health system administration representatives. The aim of this report is to describe how stakeholders were involved in research project planning and implementation with the goal of providing a set of recommendations for other researchers that are specific and practical. The present paper will serve as a guide for engaging with stakeholders in community research projects that are designed to improve patient outcomes.

The research team used the Reach, Efficacy, Adoption, Implementation, and Maintenance (RE-AIM) framework [14, 15] to inform the engagement process with the SAB and implement their feedback. In the community-academic partnership continuum [16], this work falls between the *consultative* and *collaborative* level of engagement with the SAB. This means that SAB members were not only a tremendous source of expertise but also viewed and treated as collaborators in a jointly-executed venture with the research team

## 2. Methods

### 2.1 SAB Recruitment

The RCT (individualized Texting for Adherence Building – Cardiovascular; iTAB-CV) will recruit patients with a clinical diagnosis of bipolar disorder type I or type II and hypertension (HTN). HTN will be indicated by any of the following: 1) a diagnosis of stage 1 or 2 HTN with a systolic blood pressure ≥130; 2) a diagnosis of HTN per patient self-report ≥ six months prior to enrollment; 3) prescription of at least one regularly scheduled antihypertensive medication for ≥ three months since diagnosis; and 4) self-reported poor adherence to antihypertensive medication defined as missing 20% or more of the antihypertensive medication within either the past week or past month as identified by the Tablets Routine Questionnaire (TRQ) [17]. Recruitment efforts will monitor trial enrollment to ensure participants are representative of the demographics of the target population in the trial’s geographical catchment area: 50% female, 47% Black/African American, 52% non-Hispanic White, and 1% Hispanic or Latino.

Recruitment for the Stakeholder Advisory Board (SAB) targeted patients, patients’ family members, clinical providers (e.g., psychiatrists), and health system administrative staff members. The goal was to recruit twelve SAB members: four patients with lived experience of hypertension and bipolar disorder, two family members, four clinical providers, and two health system administrative staff members. Clinical providers and health system administrative staff members were recruited first. The research team recruited clinical providers from within the Department of Psychiatry at an academic medical center. The project manager contacted clinical providers via phone and email to ascertain their interest in enrolling. Three clinical providers were contacted and enrolled. The research team identified health system administrative staff members from the Department of Psychiatry intake and administrative team. The project manager reached out to these staff members directly to invite them to participate in the SAB. A total of three administrative staff members were contacted and recruited.

Guided by the RE-AIM planning and evaluation framework [14, 15], the study team aimed to identify participants who were representative of the patient population targeted in the RCT. Patients with lived experience with bipolar disorder and HTN were identified by the research team and clinical providers. The project manager and a research assistant (RA) reached out via phone and email to potential participants. An RA also contacted potentially-eligible patient participants from prior research studies who had agreed to being contacted for future studies. A total of ten patients were contacted, and three were enrolled. Of the seven contacted patients who did not enroll as members of the SAB, the majority failed to respond rather than directly declined participants. Patients who were successfully contacted by the research team were also asked if a family member might be interested in participating in the SAB; however, this recruitment strategy did not result in any successfully recruited family members. Family members were also referred from an ongoing study focused on caregivers of people with bipolar disorder. The RA called the participants of that study to assess their interest, then referred them to enroll in the SAB. A total of three family members were contacted and enrolled into the SAB.

### 2.2 Responsibility and Expectations of the SAB

A primary role of the SAB is to assist with participant identification and recruitment for the RCT. Prior to participant enrollment in the RCT, the SAB met three times for one-hour Zoom meetings over the course of three months (January, February, and March 2020). For the remainder of the five-year RCT study period, SAB members will be expected to meet on an annual basis. The SAB members had no study-specific responsibilities outside these planned SAB meetings. However, some spontaneous conversations between the SAB members and research team occurred outside of formal SAB meetings, specifically for providing additional recommendations for participant recruitment avenues.

### 2.3 Structure of SAB Meetings and Communication

In line with best practices [4], the SAB meetings were approached with intentionality. The research team prepared a detailed agenda in advance of each meeting, and meeting minutes were taken during the calls. Due to the ongoing COVID-19 pandemic, SAB meetings occurred over Zoom and were facilitated by the Co-PIs JL and MS. The benefit of the virtual platform was that meetings could be recorded and transcribed with IRB approval. In transcripts, SAB members were de-identified and labeled consistently across the three meetings (e.g., psychiatrist 1 was always labeled as “psychiatrist 1”). For one SAB members who expressed concern about the loss of confidentiality in a group Zoom format, the RA met with that individual separately and used the same meeting agenda to collect the SAB member’s feedback. Key take-away points from each SAB meeting were summarized in an electronic document, and all SAB recommendations were considered by the research team.

### 2.4 SAB Meeting Evaluation

Web-based (Qualtrics) surveys were sent to SAB members via email following each meeting to assess SAB member satisfaction. Question development was informed by principles of effective stakeholder engagement [2-4, 18], including respect, trust, and transparency. The survey was pragmatic and included three items to avoid participant burden. Surveys were analyzed using descriptive statistics.

## 3. Results

Table 1 summarizes recruitment of SAB members, including the number of individuals who were reached and enrolled in the SAB. Recruitment efforts began three months prior to the first SAB meeting, with six members attending the first SAB meeting (two psychiatrists and three administrators). Two additional SAB members were consented for the second meeting (two patients), but one was unable to attend. Three additional members were consented for the third meeting (two family members and one patient). The first meeting was conducted prior to finishing enrollment of the SAB, and five additional SAB members were enrolled between the first and third meeting. Due to scheduling challenges, not all members could attend every SAB meeting.

**Figure 1.**
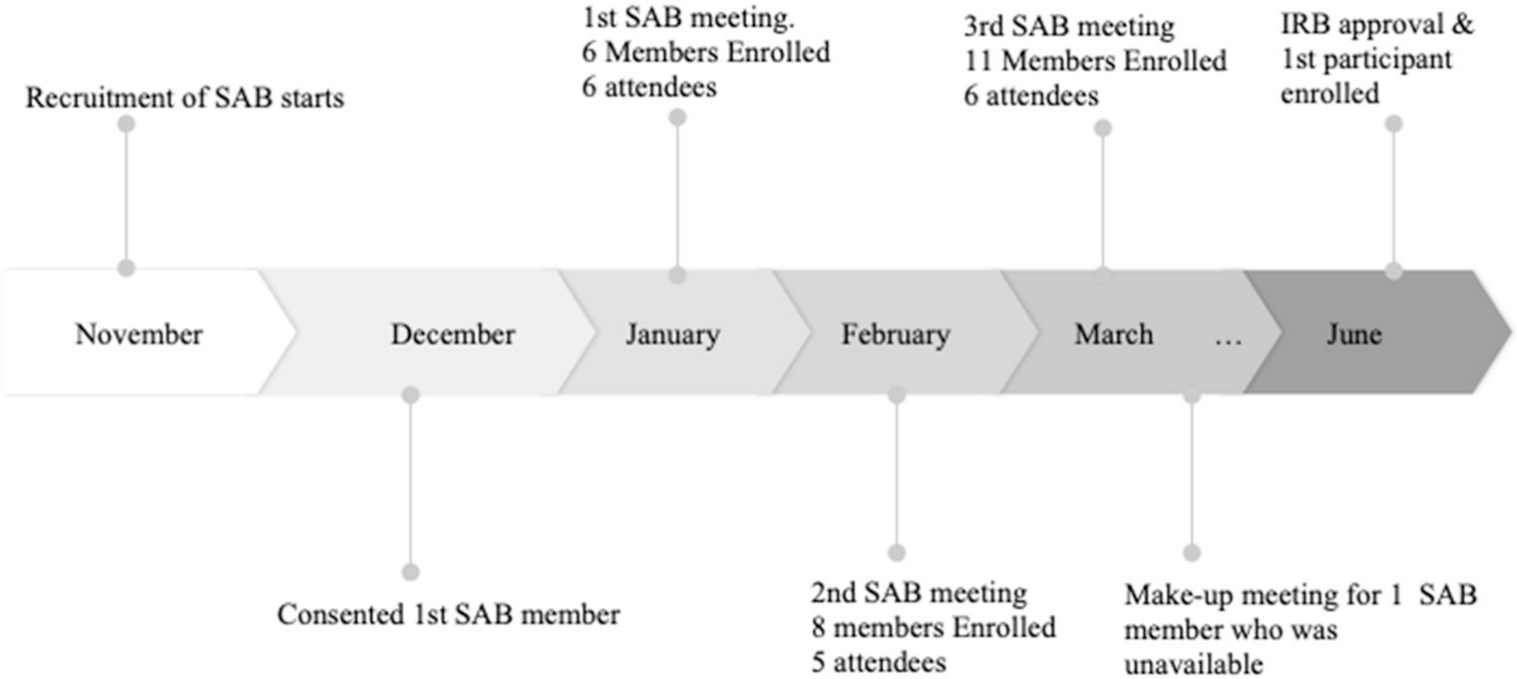
Stakeholder Advisory Board Recruitment Timeline and Attendance

Figure 1 shows the timeline of SAB recruitment and number of attendees in the meetings spanning a five-month timeline. All SAB members were recruited and the three SAB meetings were conducted prior to the start of recruitment for the RCT. The first participant in the RCT was enrolled three months after the third SAB meeting.

Table 2 shows the demographic characteristics of the SAB. Eleven SAB members were successfully recruited, and comprised of 81.8% female, 100% non-Hispanic, 36.4% Black, 54.6% White, and 9.1% Asian. Across all member types, the SAB members had, on average, 11.2 years of professional or lived experience with BD, and 7.8 years of professional or lived experience with HTN.

**Figure 2:**
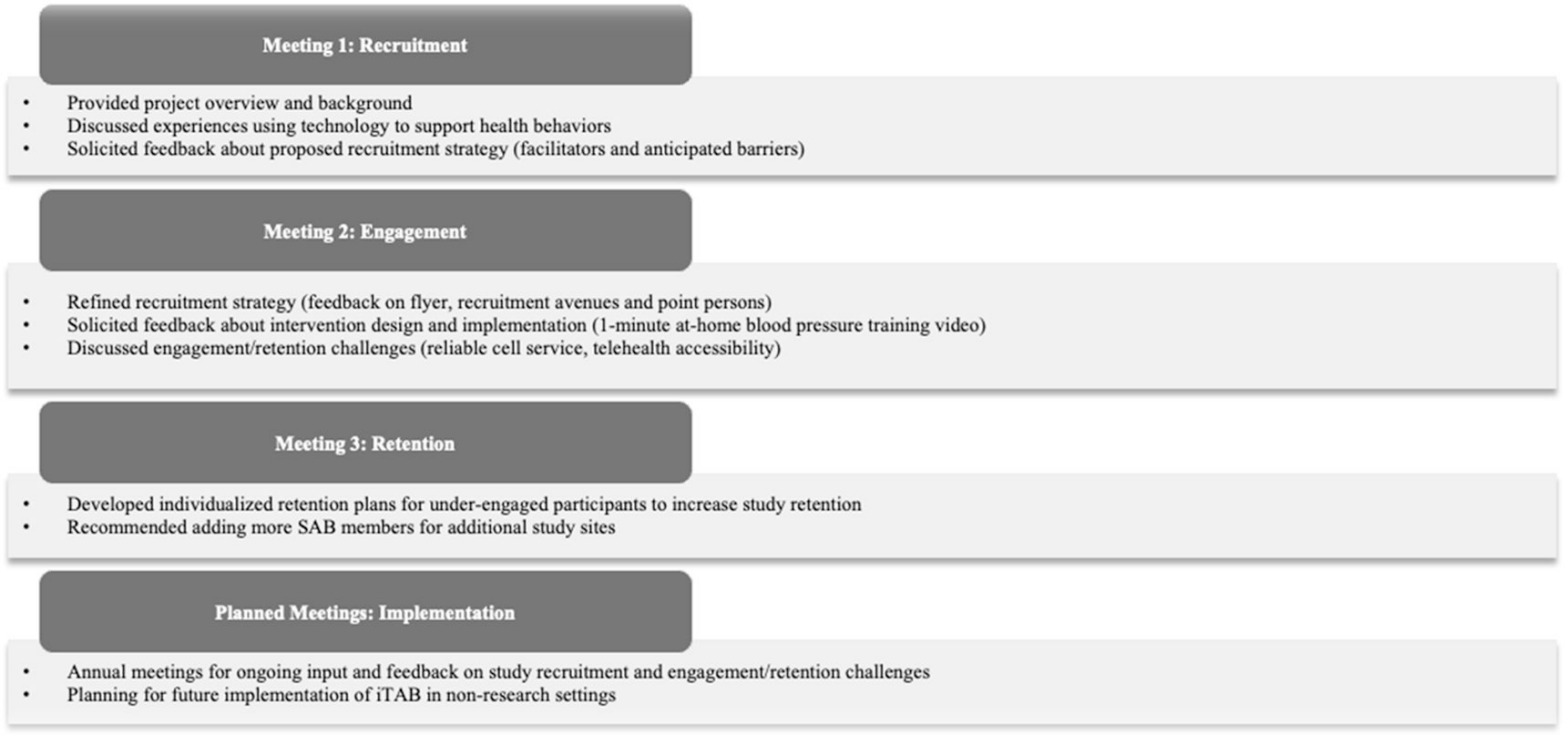
Topic and Scope of Engagement for SAB Meetings

Figure 2 summarizes the topics and take-aways from the three SAB meetings

### 3.1 Contributions of the SAB, including identification of study implementation barriers and strategies for overcoming these challenges

#### 3.1.1 Trial Design

Input from the SAB prompted the research team to reconsider the study inclusion/exclusion criteria, which originally required a confirmed bipolar disorder diagnosis two years prior to enrolling in the study. This was viewed by the SAB as being overly exclusionary and not only posed a major obstacle to recruitment but also potentially left out an important under-represented in research population of individuals who may have been unaware and untreated for bipolar disorder. The SAB’s input was instrumental in re-negotiating the inclusion criteria so that a structured interview could be used to determine eligibility for enrollment for any diagnosis of bipolar disorder (unprohibited by the length of time of diagnosis).

#### 3.1.2 Participant Recruitment

SAB members provided input for how to disseminate recruitment materials for a fully-remote study. COVID-19 was a primary motivating factor for pursuing a virtual format for assessment and health visits. Fortunately, the iTAB-CV adherence intervention was already operationalized as a fully-remote technology. One advantage of gaining approval from the IRB and the National Heart Lung and Blood Institute for the delivery of remote study assessments was that it enabled a wider recruitment pool, from a regional to a national level. However, conducting the study fully remotely introduced new challenges. For example, one of the SAB members pointed out that, traditionally, physical flyers would be displayed in the vicinity of a study or in waiting rooms, and people walking past would pull a tab and call the study coordinator if they were interested in participating. In the era of telehealth, however, recruitment is more complex and requires a coordinated online effort. The SAB provided invaluable feedback on improving the virtual flyer, which could then be disseminated more widely. The research team solicited feedback from the SAB about the appearance of the study flyer, and the SAB’s key input was to make the flyer more infographic and to include instructions about remote participation. To implement this feedback, the research team included more graphics on the flyer and incorporated a QR code with directed participants to a landing website for the research study. In addition to providing input on the design of the study flyer, the SAB also recommended that the research team engage with social workers and behavioral health workers to assist with identification of potentially-eligible study participants and recruitment.

SAB members were also willing to contribute to participant recruitment outside the SAB meetings. Following the third SAB meeting, a research assistant (RA) sent an IRB-approved email and flyer to clinical providers in the SAB requesting assistance in recruitment efforts for the RCT. Three health system staff members agreed to add a question regarding HTN to their patient intake form to assist in identifying potentially-eligible participants for the RCT. The health system administrative staff members also gave the study RA confidential access to their Psychiatry Intake REDCap data for 2020 and 2021; access to this data allowed the RA to identify patients with HTN and a diagnosis of BD who had agreed to be contacted for research studies. Only contact information was shared, other personal health information was protected to preserve confidentiality. One administrative staff member agreed to print the study flier to post it in their department and send to other administrators who they knew at main and off-site medical offices. The RA also attended the administrator’s intake team meeting to present the study to intake staff for them to pass on or advertise to potentially eligible patients.

One administrative staff member assisted in connecting study staff with a Community Based Research Network (CBRN) spanning several sites in the Cleveland area. The RA subsequently attended a meeting of the CBRN to present details of the study to clinicians and administrators and ask for their support in advertising to, and referring, potentially eligible and interested participants. The RA followed up with the CBRN coordinator and other members to announce the official beginning of recruitment for the study and again request their assistance with recruitment efforts. The CBRN coordinator agreed to circulate the flyer and include the study on their website.

#### 3.1.3 Participant Retention

The SAB also discussed the best practices for following up with under-engaged participants during different phases of the intervention. The research team hypothesized that conducting a fully remote study might potentially diminish personal connections that are typically established between study participants and the study staff, which, in turn, could potentially reduce participant engagement in a study. The SAB provided recommendations about the frequency and method of following up with participants who are under-engaged in the study in order to maximize participant retention. The SAB also suggested individualized engagement methods for retaining participants in the study, where research associates and the mobile interventionists would follow-up with under-responsive participants directly and through tangential systems of support (e.g., social workers, family members, and providers). The consensus from the SAB was that providers should ask patients directly about their preferred modalities of engagement (e.g., phone-call, text, or email). SAB members proposed following up after 8 hours and then again after 5 days of no contact. They also suggested reaching out to family and friends of participants who have been under-responsive to provide community support for individuals to remain in the research study. The SAB also suggested that the research team continue to recruit more individuals with lived experiences to serve on the SAB, as these individuals may have advice on long-term study retention.

#### 3.1.4 Patient Accessibility of Technology

The SAB played an integral role in helping the research team identify potential barriers to patient participation in the study, including engaging with the intervention (iTAB-CV) and completing the study visit assessments (baseline visit and study visits at 3, 4, 5, 6, 9, and 12 months). One patient-level factor that may limit study participation is patient access to consistent and reliable cellular service. One of the SAB members estimated that a quarter of patients would experience issues accessing and being able to use the technology associated with the intervention. Another SAB member noted that most patients have smartphones but estimated that not all of them are proficient in using them. The SAB suggested that participants work closely with the study staff to become proficient in using their smartphones during the intervention.

**Figure 3.**
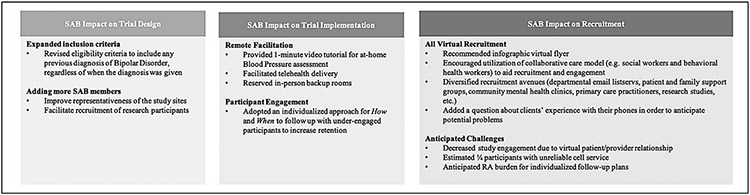
Impact of the Stakeholder Advisory Board

To facilitate a fully-remote research study, SAB members suggested that the research team provide study participants with an instructional video with information about bipolar disorder, a demonstration of how to engage with the texting messaging intervention (iTAB-CV), instructions about how to conduct at-home blood pressure monitoring, and instructions on how to use videoconferencing to participate in the study visits. The research team responded to the SAB’s recommendation by sharing with them a one-minute training video about self-administration of at-home blood pressure (BP) readings. The SAB approved the video and indicated that it was clear and helpful. Patients enrolled in the study would also receive further information about how to engage with the texting system from the mobile interventionist in a personalized session and follow up calls as needed. Figure 3 summarized the impacts of the SAB on trial design and participant recruitment and retention for the RCT

For participants who do not have access to computers or Zoom accounts, the SAB suggested that the study team set up telepsych rooms for clinicians and participants to access during the study visits. In response to the SAB’s recommendation, the research team reserved two tele-psych rooms for providers to use with participants in the study. Ongoing study efforts include reserving in-person rooms at satellite study locations. The research team additionally adopted in-person interview sessions for participants with limited technological access and/or preference for completing study visits in person. The study team also modified the ways in which participants could report their blood pressure levels including real-time on Zoom, real-time via phone call, or sending a picture of the home blood-pressure monitor screen. These modifications were driven by the goal to minimize barriers to participant engagement.

### 3.2 SAB Member Satisfaction

Satisfaction surveys were disseminated following each of the three SAB meetings. Responses to the surveys are displayed in Table 3. SAB members were provided with the link at the end of the meeting and were sent reminders to complete the surveys the following day. Ten total responses were recorded; three from the first meeting, three from the second meeting, and four from the third meeting. Based on the timestamps of collected survey data, SAB members either responded to the surveys immediately following the meetings or not at all. Across meetings, surveys indicated that all SAB members strongly agreed or agreed that the objectives of the meetings were clear (60% strongly agree), that they were able to participate in the discussions (90% strongly agree), and that they were heard (90% strongly agree).

## 4. Discussion

The purpose of the ongoing trail’s SAB is to reach, recruit, and retain a representative sample of hypertensive persons with bipolar disorder. The questions posed to the SAB during SAB meetings focused on the RE-AIM dimension of reach, defined as the absolute number, proportion, and representativeness of individuals who are willing to participate in a given intervention. Input from key stakeholders (patients, patient family members, providers, and health administrative staff) informed the planning and implementation of study procedures to promote the reach of the intervention in an equitable fashion. Significant suggestions from the SAB included expansion of participant inclusion/exclusion criteria and helping the research team operationalize a fully remote/virtual research study. The SAB significantly contributed to identifying strategies for participant recruitment and retention, as well as consideration of patient-level barriers to study participation (e.g., access to technology such as videoconferencing and uninterrupted access to cellular service with text message abilities). Throughout our initial SAB meetings, SAB members felt that the objectives of the meetings were clear, they were able to participate in the discussions, and they were heard. Our study indicates feasibility of engaging SABs in clinical research, which improved our intervention design and may lead to improved participant engagement.

Despite the advantages of having a SAB, several important challenges should be considered, including privacy and confidentiality concerns, technological challenges, and scheduling difficulties. For example, one of the family members invited to join the SAB was concerned about the loss of privacy and confidentiality. The research team thus offered to meet with this individual separately to ask questions that were being asked of the broader SAB group, but in a setting that would be more private and comfortable. The individual agreed and a separate interview was conducted in addition to the three primary SAB meetings. Accommodations like this must be considered in advance of engaging in community-driven research. Another challenge arose when a prospective SAB member experienced difficulty accessing the eConsent form and completing the consent process. The individual was sent the eConsent link directly twice; both were received but neither could be opened on the participant’s end, thus the participant was not enrolled. Additional technical support and problem solving is needed to equitably sustain the participation of stakeholders from the community. Scheduling was a third challenge, and research teams should anticipate difficulty in identifying meeting times in which all SAB members can attend. The first SAB meeting was scheduled prior to completing recruitment of the entire SAB, and the following meetings were planned to occur at one- and two-month intervals after the first meeting. There may have been increased consensus on feedback or more insights to offer the study team had there been the same SAB members present across the three meetings. Furthermore, while introductions of new members were made, in the service of not repeating the same information, there was not as much background material about the study presented in meetings two and three. Thus, the newer members might have felt less informed and engaged. We recommend that future studies attempt to finish recruiting their SAB members prior the first meeting and then consult the SAB on the meeting schedule to determine if, perhaps, more frequent meetings would be helpful. A drawback of finishing SAB recruitment before scheduling is potentially delaying the start of the study as well as diminished enthusiasm from SAB members who agreed to participate first. Because it may be unrealistic to finish recruitment of all SAB members prior to the first meeting, we recommend alternative solutions such as having mini-meetings between the primary SAB meetings to help bring newly-recruited members up to speed prior to the larger meetings.

### 4.1 Lessons Learned

Findings from this study support the existing literature on best practices of stakeholder engagement in clinical research (Barger et al 2019). For example, early and continual involvement of the SAB was particularly useful. At the first meeting, it was essential to provide all SAB members with a brief overview of the RCT and discuss the life cycle of the clinical trial. This made it possible for stakeholders to identify potential challenges early on for the research team to address. Hosting more frequent meetings prior to the start of the RCT was also helpful for the research team to build a stronger relationship with the SAB, with open and honest communication to foster meaningful collaboration going forward. In these early meetings, it was essential that the research team posed questions to the SAB that were actionable (e.g., asking the SAB for feedback on recruitment materials and recruitment pathways). SAB feedback could then immediately be incorporated to adapt the intervention and its implementation. We recommend sharing meeting agendas in advance with the SAB in order to clarify the meeting objectives. Finally, a fundamental feature of community-based participatory research is to report back to stakeholders on how their feedback is utilized and demonstrate any products or changes that result from their feedback [19].

### 4.2 Impact of COVID-19

Recruitment of SAB members was impacted by the ongoing pandemic, COVID-19. As patients were not attending in-person clinical visits, they were unlikely to see posted flyers. As such, most recruitment occurred virtually via phone calls, which made it more difficult to recruit family members and patients. Engagement with the SAB was entirely virtual as well, through Zoom. This made meetings easier to attend for some SAB members, who could join right after work and not have any commute or transportation concerns. Completion of the satisfaction surveys after meetings may have been lower than compared to in-person collection.

The ongoing COVID-19 pandemic impacted relatively few but significant features of the study. Fortunately, since the SAB was always intended to meet virtually this aspect of the study remained unaffected by the pandemic. One primary change was adjusting elements of the study design from in-person to virtual, specifically for at-home blood pressure monitoring and research telehealth visits. Secondly, the research team communicated almost entirely via remote methods, which is progressively improving over time but still cumbersome. The most significant consequence is that people who are vulnerable to COVID-19 (i.e., those with serious mental illness, and racial groups such as African-Americans who are more likely to have HTN) are less likely to seek any healthcare, including involvement in research studies.

### 4.3 Best Practices for Engaging SABs

Our recommendations for meaningful engagement with SABs are comparable to the NIH guidelines around stakeholder engagement, as well as leading implementation Science guidelines [3, 4, 20, 21]. Our recommendations for best practices for future research are summarized in Table 4. The Inverse Care Law and Inverse Equity Hypothesis put forth by Cookson et al (2021) posits that individuals with the greatest need of clinical intervention are generally the least likely to receive it; engagement with a SAB is one strategy of addressing care gaps, including representativeness of patient groups in clinical research.

## Figures and Tables

**Table 1:** Stakeholder Advisory Board Recruitment and Meeting Attendance

SAB Member Category	Admin	Clinicians	Family Members	Patients	Total
**Recruitment**					
Reached	3	3	3	10	19
Enrolled	3	3	2	3	11
Other*			1	7	8
**Meeting attendance**					
Meeting 1	3	3	2	3	6
Meeting 2	3	3	0	2	5
Meeting 3	3	3	2	3	6

* Note: Other indicates potential participant did not respond (n=3), not interested (n=2), unable to participate (n=2), and not eligible (n=1).

**Table 2:** Demographics and Characteristics of iTAB SAB Members

SAB Role	Clinician (N=3 27.3%)	Hospital Administrator (N=3 27.3%)	Patient (N=3 27.3%)	Family Member (N=2 18.2%)	Total SAB Participants (N=11)
**Age (in years) Mean (SD)**	50.0 (9.5)	49.0 (16.4)	51.5 (19.1)	66.5 (20.5)	53.30 (14.7)
**Gender % (N)**					
Male	18.2% (2)	0.0% (0)	0.0% (0)	0.0% (0)	18.2% (2)
Female	9.1% (1)	27.3% (3)	27.3% (3)	18.2% (2)	81.8% (9)
**Education Completed (in years) Mean (SD)**	21.7 (1.5)	15.7 (4.7)	15.3 (3.1)	^†^12.0	17.0 (4.4)
**Race % (N)**					
White/Caucasian	18.2% (2)	18.2% (2)	9.1% (1)	9.1% (1)	54.6 % (6)
Black	0.0% (0)	9.1% (1)	18.2% (2)	9.1% (1)	36.4% (4)
Asian	9.1% (1)	0.0% (0)	0.0% (0)	0.0% (0)	9.1% (1)
**Ethnicity % (N)**					
Non-Hispanic	27.3% (3)	27.3% (3)	27.3% (3)	18.2% (2)	100.0% (11)
**Years of lived/ professional experience w/ bipolar disorder Mean (SD)**	12.0 (10.6)	10.0 (17.3)	8.3 (2.1)	16.0 (5.7)	11.2 (9.7)
**Years of lived/ professional experience w/ hypertension Mean (SD)**	15.3 (8.1)	3.3 (5.8)	5.7 (0.6)	6.5 (7.8)	7.8 (7.1)

Notes: SD= standard deviation

† Only one family member provided their years of education

**Table 3.** Stakeholder Advisory Board Survey Responses

SAB Satisfaction Survey Response (N=10)	
The agenda/objectives for the meeting were clear.	
Strongly agree	60% (6)
Agree	40% (4)
I had the opportunity to participate in the discussion and share my perspective.	
Strongly agree	90% (9)
Agree	10% (1)
I felt heard.	
Strongly agree	90% (9)
Agree	10% (1)

**Table 4.** Best Practices of Stakeholder Engagement in Clinical Research for Severe Mental Illness

Best Practices of SAB Engagement in Clinical Research
**Early and Continual Communication** More frequent meetings at the beginning builds stronger, more collaborative relationships Make accommodations when needed (e.g., scheduling)
**Clear Description of Study Design and Timeline** Ensures all stakeholders have equal understanding of the topic in order to freely contribute ideas Makes it possible for stakeholders to identify challenges early on
**Clarify SAB Role and Expectations** Cultivates mutual respect and understanding between SAB and research team
**Actionable Requests During Meetings** Feedback can then be immediately incorporated into the intervention and/or its implementation
**Share Meeting Objectives** Keeps SAB meetings focused and improves meeting evaluations Enables SAB members to prepare in advance
**Report Back** Shows the SAB that their input is valued, making it more likely they will continue contributing
